# Reversal of threatening blindness after initiation of eculizumab in Purtscher-like retinopathy secondary to atypical hemolytic uremic syndrome

**DOI:** 10.1007/s10792-017-0470-1

**Published:** 2017-03-08

**Authors:** J. E. Ramos de Carvalho, R. O. Schlingemann, M. Oranje, F. J. Bemelman, M. J. van Schooneveld

**Affiliations:** 1 Department of Ophthalmology, Academic Medical Centre, University of Amsterdam, Amsterdam, The Netherlands; 2 Division of Internal and External Medicine, Department of Nephrology, Academic Medical Centre, University of Amsterdam, Amsterdam, The Netherlands

**Keywords:** Atypical hemolytic uremic syndrome, Complement, Eculizumab, Purtscher-like retinopathy

## Abstract

Purtscher-like retinopathy, a rare manifestation of systemic thrombotic microangiopathy, is a potentially visually debilitating condition with no effective proven treatment. Distinct pathogenic pathways have been proposed as etiological factors. We revisit the etiology of Purtscher-like retinopathy based on the rapid response and profound visual improvement after initiation of systemic intravenous eculizumab, an inhibitor of the complement cascade, in a patient with Purtscher-like retinopathy secondary to familial atypical hemolytic uremic syndrome (aHUS) due to a mutation in complement factor H. We hypothesize that the efficacy of eculizumab in this patient provides evidence for pathogenic events in the retina similar to those encountered in the renal microvasculature of aHUS patients, namely complement-mediated thromboembolization as a result of activation of the complement cascade in endothelial cells with release of tissue factor and development and amplification of a procoagulant state. To the best of our knowledge, this is the first report in the literature of eculizumab as an effective therapeutic strategy in Purtscher-like retinopathy.

## Introduction

Purtscher and Purtscher-like retinopathy are rare, often bilateral, multifactorial downstream occlusive thromboembolic retinopathies, mediated in a large proportion of cases by uncontrolled complement activation. The designation Purtscher retinopathy implies a traumatic etiology, whereas Purtscher-like retinopathy depicts non-traumatic causes [1].

Several mechanisms have been brought forward as hypothetical triggers of Purtscher and Purtscher-like retinopathy [1–3]. Although the pathogenesis is likely multifactorial and differs according to the underlying predisposing condition, embolization of the retinal microcirculation has been proposed as the common pathogenic ground for the retinal findings [4–7]. Potential sources of toxic and/or obstructive emboli include air, fat, fibrin, platelet and leukocyte aggregates [1], emboli arising from orbital steroid injection [8] and retrobulbar anesthesia [8–11], and amniotic fluid emboli after childbirth [12]. Uncontrolled complement activation has been proposed to play a major role in the pathogenesis of Purtscher-like retinopathy by mechanisms involving endothelial damage and activation of the clotting cascade [5, 6, 13, 14] and development of leukocyte and platelet aggregates [1, 4]. Other proposed mechanisms include intravascular volume surge such as in sudden expansion of retinal veins [1], hyperviscosity [15], intracephalic shock waves such as in sudden increase in intracranial pressure with resulting precapillary occlusion at the level of the lamina cribrosa [16], capillary endothelial damage [17] and vascular endothelial dysregulation and ensuing endothelin-induced vasculopathy [16]. Associated systemic entities include acute pancreatitis [18–25], pancreatic adenocarcinoma [26], systemic lupus erythematosus [27], renal failure [28, 29], amniotic fluid embolization [12], thrombotic thrombocytopenic purpura [30–33], hemolytic uremic syndrome (HUS) [31, 34] and cryoglobulinemia [35–37]. In specific disorders, the diagnosis of Purtscher-like retinopathy accompanies multisystem organ failure and therefore portends a poor prognosis [38].

Patients present with sudden visual acuity loss of variable severity, hours to days after the onset of the associated etiology. Some patients may be asymptomatic which likely results in underreporting [4]. The majority of cases (83–92%) show funduscopic evidence of retinal nerve fiber layer infarcts (cotton-wool spots) and intraretinal hemorrhages [1, 2]. Purtscher “flecken” corresponds to areas of intraretinal whitening with a clear zone (within 50 μm) on either side of the retinal arterioles, venules and precapillary arterioles. These lesions result from precapillary arteriolar occlusion and, albeit pathognomonic, can be identified in only 50% of cases [2]. Other less frequent findings include macular edema, optic disk swelling and a pseudo-cherry red spot. Fluorescein angiography shows evidence of an occlusive thromboembolic retinopathy with areas of retinal non-perfusion and leakage of dye from retinal arterioles, capillaries, venules and optic disk [4].

Up to 40% cases may be associated with normalization of all retinal findings and restoration of normal visual acuity; however, a significant proportion of patients develop optic atrophy, mottling of the retinal pigment epithelium (RPE), retinal thinning and narrowing of retinal arteries [4, 39]. The prognosis for the individual patient is difficult to predict due to the lack of clear and validated predictors. Treatment strategies range from watchful waiting, plasmapheresis or high dose systemic corticosteroids [2, 40–42].

Hemolytic uremic syndrome is characterized by non-immune hemolytic anemia, thrombocytopenia and renal impairment. Most cases (90%) are secondary to infection with Shiga toxin-producing bacteria as well as other bacteria, such as *Streptococcus pneumoniae* [43]. Non-infectious causes, classified as atypical hemolytic uremic syndrome (aHUS), are linked to uncontrolled complement activation. The familial type of aHUS has a particularly poor prognosis, with progression to end-stage disease occurring in 50 and 80% of cases [44]. Purtscher-like retinopathy has been reported to occur in a minority of patients with HUS [45–47].

We present a case of Purtscher-like retinopathy secondary to aHUS due to complement factor H (*CFH*) mutation and resulting complement overactivation, successfully treated with systemic intravenous administration of eculizumab (Soliris; Alexion Pharmaceuticals, Cheshire, CT, USA), an inhibitor of terminal complement activation. We hypothesize that eculizumab may be an alternative therapeutic strategy for severe Purtscher-like retinopathy associated with deregulated activation of the complement pathway. Based on the response achieved after initiation of treatment, an alternative etiology for Purtscher-like retinopathy is proposed after a short review of the literature.

## Case description

A 20-year-old Caucasian woman presented with complaints of subacute painless loss of vision of her left eye. She was referred to our clinic for intravitreal ganciclovir treatment after a putative diagnosis of bilateral cytomegalovirus retinitis had been made by an ophthalmologist at her local hospital the previous day. She was one of three sisters with familial aHUS due to a missense mutation (c.3572 C > T, Ser1191Leu) in exon 23 of Complement Factor H (*CFH* gene). At 6 years of age, she underwent bilateral nephrectomy followed 1 year later by a living donor renal transplant. After 1 year, she developed a systemic cytomegalovirus infection with relapse of aHUS. Her immunosuppression was tapered to a calcineurin-free regimen, and chronic maintenance plasmapheresis was started. Four years later, a transplant biopsy showed chronic allograft nephropathy with global glomerulosclerosis and tubular atrophy. There were no treatment options other than supportive care and her renal function slowly declined hereafter. At 19 years of age, she reached end-stage renal failure, immunosuppression was further tapered and she was started on dialysis. The maintenance plasmaphereses were halted. During dialysis, she complained of seeing “black spots” and was referred to a local ophthalmologist who in turn referred her to our service. Her family history included two sisters, one of them her identical twin, with aHUS and *CFH* mutation. Her brother was an asymptomatic carrier for the *CFH* mutation, and her younger sister was not affected. She had no past history of ocular illnesses.

**Fig. 1:**
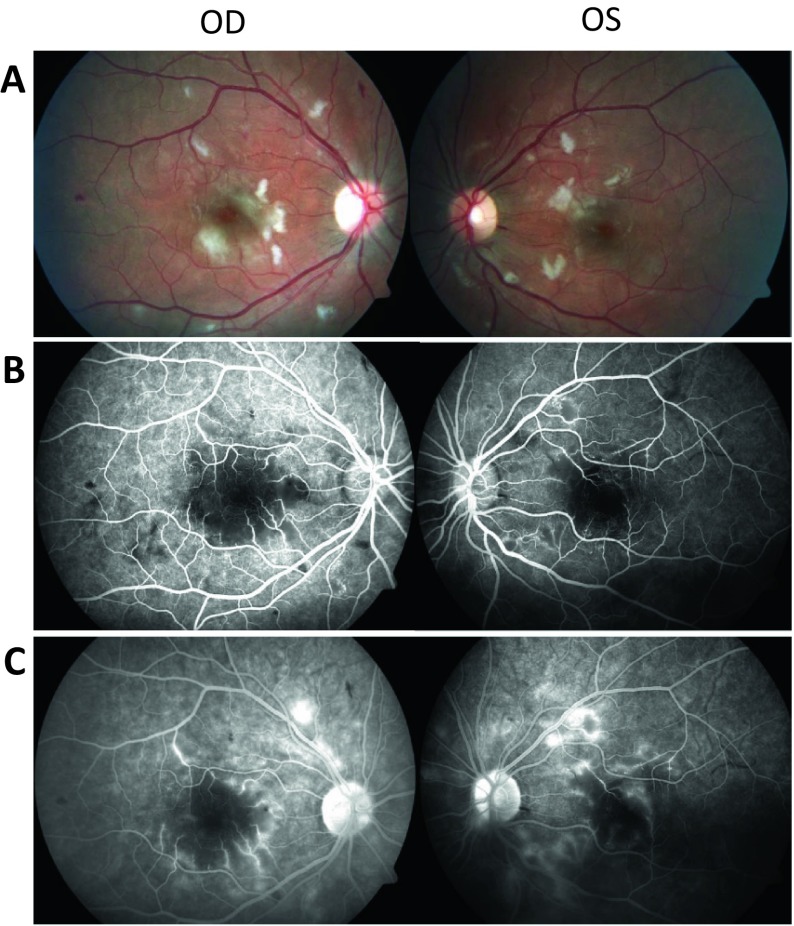
Retinal photographs and fluorescein angiogram appearance at initial presentation. **a** Fundus appearance at presentation. Note the diffuse retinal nerve fiber layer infarcts, inner retinal ischemia and scattered retinal hemorrhages. Visual acuity was 20/15 and 20/200^−2^ in the right and left eye, respectively. **b** Mid-phase intravenous fluorescein angiogram of the same patient at initial presentation. Note the parafoveal and perifoveal areas of capillary obstruction and retinal ischemia correspondent to the areas of inner retinal ischemia seen in the fundus picture. **c** Mid- to late-phase fluorescein angiogram in the same patient. Note the parafoveal pericapillary staining and mild macular edema with late leakage in the left eye. Angiographic findings were mostly confined to the macula and immediately nasal to the optic disk

**Fig. 2:**
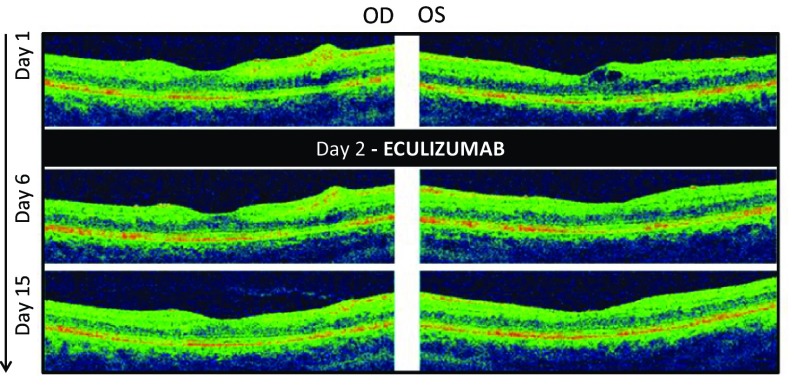
Optical coherence tomography findings at initial presentation and at day 6 and 15. Inner retinal ischemia was evident in both eyes, corresponding to the cotton-wool spots demonstrated in the color photographs. The right eye, with normal visual acuity at presentation, had no evident macular edema. The left eye, with severe visual acuity loss, had mild cystoid macular edema which regressed rapidly after eculizumab administration. At day 15, visual acuity in the left eye had improved significantly alongside resolution of the macular edema and inner retinal ischemia

On ophthalmologic examination, best corrected visual acuity was 20/15 and 20/200^−2^ in the right and left eyes, respectively. There was no relative afferent pupillary defect. Intraocular pressure was normal. The anterior chambers and vitreous were clear in both eyes. Fundus examination revealed cotton-wool spots and mild flame-shaped hemorrhages in both eyes. The left eye showed mild macular cystoid macular edema. The retinal vessels were of a normal caliber in both eyes. An inconspicuous choroidal nevus was present in the nasal retina of the right eye. Fluorescein angiography revealed early hypofluorescence, areas of non-perfusion, capillary obstruction and dropout, inner retinal ischemia in the parafoveal and perifoveal zone corresponding with the cotton-wool spots and perifoveal capillary leakage in the late frames. The left eye showed mild cystoid macular edema nasal to the fovea (Figs. 1, 2). Mild peripapillary leakage was noted in the late frames of the left eye. Besides a small hemorrhage peripherally, all other angiographic findings were confined to the posterior pole, namely within the macula and immediately nasal to the optic disk. Normal fluorescein transit times were observed. The degree of macular ischemia and perifoveal capillary dropout was more pronounced in the left eye in keeping with the objective visual acuity loss in that eye. These clinical findings, together with her medical history, were consistent with Purtscher-like retinopathy. At the time of referral to our clinic, she was awaiting a second living donor renal transplantation. The ophthalmic findings provided evidence that the hemolytic uremic syndrome was undertreated and indirect evidence that she had uncontrolled systemic complement activation. In order to prevent further progression of her retinopathy, and due to the severe loss of vision in her left eye, treatment with eculizumab was initiated. Eculizumab, a C5 complement inhibitor, has been praised as highly effective in the treatment of hemolytic uremic syndrome, also after transplantation. After 4 months, while on chronic eculizumab therapy, a transplantectomy was performed followed by a second renal transplantation. Both procedures proceeded uneventfully.

**Fig. 3:**
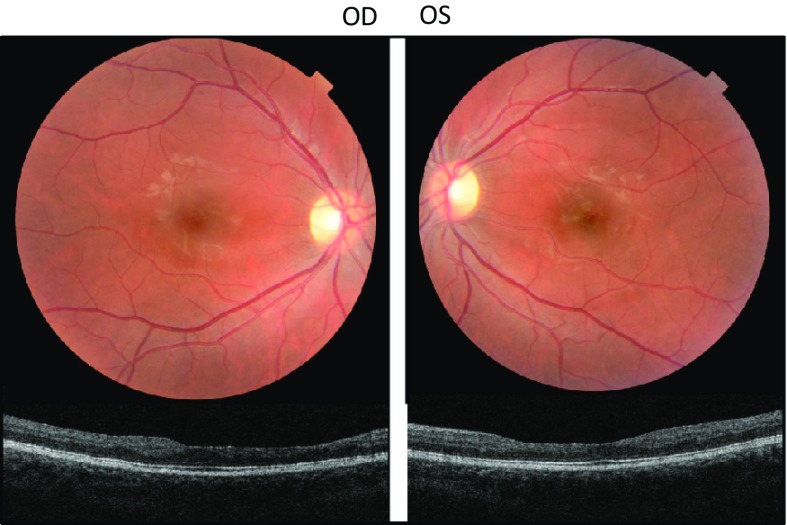
Retinal photographs and optical coherence tomography 18 months after initial presentation. *Color photographs* of the right and left maculae show a normal appearance, albeit a significant thinning is evident on optical coherence tomography. The patient is asymptomatic and has normal visual acuity (20/15 in the right eye and 20/20/15^−2^ in the left eye)

On follow-up, she reported a steady continuous improvement of her visual acuity. Five days after eculizumab administration, best corrected visual acuity was stable in her right eye and pinhole visual acuity improved to 20/50^+2^ in her left eye. Fundus examination revealed a decrease in the size of the cotton-wool spots, and optical coherence tomography showed total resolution of the cystoid macular edema in the left eye (Fig. 2). On the 14th day after treatment, she reported total resolution of all her previous visual complaints. Best corrected visual acuity was stable in her right eye and had improved to 20/15^−2^ in her left eye. Fundus examination revealed an improvement of all clinical findings, including the nerve fiber layer infarcts, flame-shaped hemorrhages and macular edema. She was referred back to her local ophthalmologist. At 18 months, visual acuity remained stable and no visual complaints were reported (Fig. 3).

## Conclusions

**Fig. 4:**
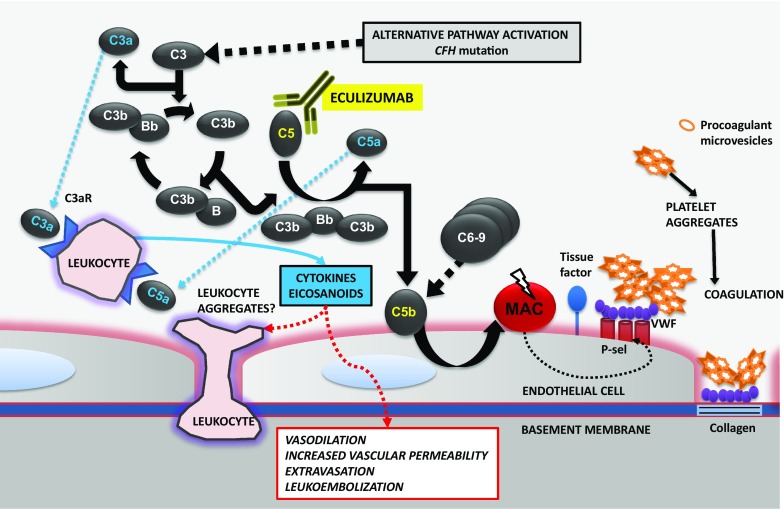
Proposed pathogenic mechanisms of Purtscher-like retinopathy secondary to uncontrolled complement activation. The alternative pathway activates formation of C3b by C3; C3b interacts with factor B which is subsequently cleaved by factor D to form the alternative pathway C3 convertase (C3bBb). This enzyme complex is covalently attached to the target via C3b while Bb is the catalytic serine protease subunit. Factor H regulates the alternative pathway by preventing the interaction of C3b with factor B, interacting with factor I-mediated cleavage of C3b and dissociating the C3 convertase of the alternative pathway (not shown). Unchecked, the terminal complement pathway is then activated with release of the complement anaphylatoxin C5a and formation of the membrane attack complex (MAC). Eculizumab binds C5 which is then unable to enter the C5 convertase (C3bBbC3b), impeding cleavage into the effector molecules, C5a and C5b, and assembly of the terminal complement complex (C5b-9). This leads to exocytosis of adhesion molecules [P-selectin (P-sel)] and von Willebrand factor (VWF) which in turn lead to platelet adhesion and aggregation, expression of tissue factor and activation of the coagulation cascade. Cell detachment exposes the subendothelial matrix, facilitating binding of VWF to collagen with ensuing amplification of the coagulation state. Platelet aggregates release procoagulant microvesicles that contain tissue factor. Receptors for C3a and C5a [C3a receptor (C3aR) and C5a receptor (C5aR)] in polymorphonuclear leukocytes and monocytes, bind these anaphylatoxins and enhance the release of cytokines and eicosanoids contributing to an increase in vascular permeability, vasodilation, leukocyte extravasation and potentially formation of leukocyte aggregates

This single case describes the favorable visual outcome achieved after systemic treatment with eculizumab in a patient with severe Purtscher-like retinopathy secondary to familial aHUS and *CFH* mutation. In our view, the retinal findings were secondary to uncontrolled systemic complement activation and as a result complement-mediated leukoembolization and/or complement activation of the coagulation cascade in endothelial cells with ensuing development of a prothrombotic state (Fig. 4).

Leukoembolization as a cause of embolic retinal occlusion has been proposed as a potential pathogenic mechanism in Purtscher-like retinopathy [6, 48, 49]. However, recent experimental studies suggest endothelial activation of the coagulation cascade by complement with formation of microthrombi as the most plausible mechanism in the pathogenesis of aHUS [50]. Likewise, we propose this can be extrapolated not only to our case, but to all cases of complement-driven Purtscher-like retinopathy. As previously mentioned, the patient was known to carry a mutation in the *CFH* gene which codes for a serum protein that regulates the alternative pathway of the complement system in the fluid phase as well as on host cell surfaces by binding through C3b and glycosaminoglycans via its C-terminal domain [51]. Mutations in the *CFH* gene of patients with aHUS are usually heterozygous in nature and cluster in C-terminal domain 19–20. This particular mutation, also found in this patient, results in normal levels of a folded, abnormal protein, that is unable to bind and regulate complement on host cells and platelets. Animal studies have confirmed development of aHUS with high C3 plasma levels in mice lacking the C-terminal end domain of factor H [52]. In contrast, complete *CFH* knockout mice develop a different renal disease pattern, namely membranoproliferative glomerulonephritis [53]. These studies support the evidence that the mutant *CFH* present in this patient is unable to bind and control complement activation on the glomerular endothelium, basement membrane, platelets and the retinal endothelium, with subsequent development of a procoagulant state that in turn resulted in the development of aHUS and Purtscher-like retinopathy.

Eculizumab, a monoclonal antibody directed against complement protein C5, prevents activation of the terminal complement cascade and the generation of effector molecules C5a and C5b-9. The ability of eculizumab to suppress complement activity in native and transplanted kidney has revolutionized the care of patients with aHUS [54–56]. Without attenuation of complement activity, either through plasmapheresis, kidney–liver transplant (*CFH* and other complement factors are produced in the liver), or eculizumab, disease recurrence occurs in approximately 80% of *CFH* mutation carriers after renal transplantation since an isolated kidney allograft does not correct the underlying genetic defect [57].

We hypothesize that complement inhibition by means of eculizumab triggered the rapid resolution of clinical findings and dramatic restoration of visual acuity in this patient. We consider it unlikely that an expectant management or other treatment options such as dosage increase of systemic corticosteroids would have resulted in a similar outcome. Nevertheless, we are aware of the limitation of assuming efficacy based on results obtained with only one patient. Confirmation of clinical efficacy requires validation from a larger clinical study. In this patient, plasmapheresis was considered as a possible treatment option; however, this would not have had an effect on complement activation which would have resulted in perpetuation of renal and (possibly) retinal findings. Furthermore, the therapeutic effect of systemic corticosteroids in severe Purtscher-like retinopathy is inconsistent, with most studies confirming no differences in visual outcome [1, 4]. Interestingly, this patient developed Purtscher-like retinopathy while on systemic corticosteroids, albeit at a low dosage. It has been suggested that corticosteroids may be inefficient in cases of Purtscher-like retinopathy that are primarily triggered by thrombotic microangiopathy, such as in aHUS [58]. Solely targeting the inflammatory component will not affect microembolization since other complex pathogenic events such as hemostasis, thrombosis and complement dysregulation will remain unanswered [58].

Several clinical parameters support the favorable effect of eculizumab in this particular patient. First, pronounced improvement of visual acuity occurred rapidly after initiation of eculizumab (20/200^−2^ visual acuity at time of diagnosis improving to 20/15, 8 Snellen lines improvement, 14 days after initial examination). Early resolution of clinical findings is associated with better final visual outcomes [2]. Evidence shows spontaneous visual improvement of at least 2 Snellen lines is likely to occur in half of cases; however, such improvement occurs mostly in patients with better visual acuity at presentation, whereas poor visual acuity at presentation, such as observed in this patient, is regarded as a poor prognostic sign for visual improvement [2]. Secondly, this patient had other established poor prognostic criteria [4], namely female gender and intraretinal macular edema at presentation.

Severe visual loss in Purtscher and Purtscher-like retinopathies is likely secondary to macular edema [4]. Moreover, the duration of retinal changes is the most important parameter for full recovery of vision and prevention of secondary development of retinal pigment epithelium and retinal nerve fiber layer atrophy with subsequent loss of differentiation between retinal layers [59]. Therefore, early reduction of macular edema should be a priority in order to prevent progression to end-stage degenerative changes.

Mutations in complement-associated genes, and particularly in the *CFH* gene, have been shown to be associated with age-related macular degeneration (AMD), one of the leading causes of blindness worldwide [60]. Several mutations in complement-associated genes reported in patients with aHUS patients, such as Arg1210Cys in *CFH,* were found to confer a high risk of AMD development [61]. The relevance of this shared genetic association between two distinct clinical phenotypes remains unknown. Retrospective analyses of AMD databases did not reveal a higher incidence of renal disorders in carriers of complement gene mutations known to cause both aHUS and AMD [62]. On the other hand, patients afflicted with membranoproliferative glomerulonephritis type 2 (MPGN2), a renal disease that is also associated with *CFH* mutations, show AMD-like features [63]. This suggests compound (genetic or environmental) factors may influence the final clinical outcome. Indeed, AMD and MPGN2 share a common pathogenic mechanism, i.e., the deposition of complement-containing material beneath the retinal pigment epithelium in AMD and along the glomerular basement membrane in MPGN2 [63]. On the other hand, the pathogenic mechanisms causative of Purtscher-like retinopathy are shared with those of aHUS, namely endothelial injury, activation of the coagulation cascade and ultimately thromboembolic microangiopathy [52]. Moreover, aHUS patients may carry multiple complement factor gene mutations, which could imply different cellular pathogenic mechanisms [64]. Cross-phenotype studies are required in order to understand similarities and differences in complement-mediated pathogenic mechanisms of both aHUS and AMD. This could elucidate the effects of eculizumab and other complement-modulating agents in the treatment of AMD and other ophthalmic diseases. A recent phase 2 study demonstrated that eculizumab failed to arrest progression of geographic atrophy (GA) when administered for 24 weeks to patients with dry AMD (*COMPLETE* study) [65]. It is likely that, although complement dysregulation is knowingly associated with AMD, pathogenesis and progression of GA may occur independently of complement overactivation [66]. In our case report, uncontrolled complement activation had a direct effect as a facilitator and trigger of the retinal thromboembolic microangiopathy, and therefore, it is highly probable that local complement inhibition by systemic eculizumab enabled the rapid resolution of the retinal microembolization with ensuing resolution of the intraretinal macular edema. These complement-mediated effects differ from those attributed to the complement pathway in diseases characterized by deposition of extracellular material such as AMD and MPGN2. Complement inhibition has been demonstrated to occur within 1 h after administration of eculizumab [55]. Therefore, eculizumab may be more efficacious in suppressing acute complement-mediated changes such as thromboembolic microangiopathy of retinal and renal vessels, as opposed to chronic changes induced by uncontrolled complement activation such as in AMD. The authors of the *COMPLETE* study argue that intravitreal administration of eculizumab might have resulted in a more favorable outcome. Our case provides indirect evidence that systemic eculizumab, at the administered dosage, does reach therapeutic concentrations at the level of the choroid and is able to penetrate the RPE and retina. In addition, the dosing regimen used for treatment of aHUS has been shown to reach drug complement-inhibiting concentrations in the peripheral blood [56].

The description of this case raises the possibility that eculizumab may be a valid therapeutic strategy in severe cases of Purtscher-like retinopathy in which the primary pathogenic trigger is attributed to dysregulated complement activity. The benefit of intervention over expectant management can only be convincingly assessed with a randomized clinical trial, which, given the rarity of the disease and the extreme high cost of the drug (about US $350.000 per patient per year), is unlikely. The dramatic and rapid visual recovery demonstrated in this patient, even in the presence of established poor prognostic signs, namely poor visual acuity at presentation, intraretinal macular edema and female gender, provides convincing evidence that eculizumab was causally related to the attained superior outcome. To the best of our knowledge, this is the first report in the literature of eculizumab as a potential therapeutic strategy in Purtscher-like retinopathy.
